# Sympathetic Nervous System and Atherosclerosis

**DOI:** 10.3390/ijms241713132

**Published:** 2023-08-23

**Authors:** Yutang Wang, Jack Anesi, Michelle C. Maier, Mark A. Myers, Ernesto Oqueli, Christopher G. Sobey, Grant R. Drummond, Kate M. Denton

**Affiliations:** 1 Discipline of Life Science, Institute of Innovation, Science and Sustainability, Federation University Australia, Ballarat, VIC 3350, Australia; 2 Cardiology Department, Grampians Health Ballarat, Ballarat, VIC 3350, Australia; 3 School of Medicine, Faculty of Health, Deakin University, Geelong, VIC 3216, Australia; 4 Centre for Cardiovascular Biology and Disease Research, Department of Microbiology, Anatomy, Physiology & Pharmacology, School of Agriculture, Biomedicine & Environment, La Trobe University, Melbourne, VIC 3086, Australia; 5 Department of Physiology, Monash University, Melbourne, VIC 3800, Australia; 6 Cardiovascular Disease Program, Monash Biomedicine Discovery Institute, Monash University, Melbourne, VIC 3800, Australia

**Keywords:** alpha blocker, beta blocker, blood vessel, sympathetic activity, atherosclerosis, renal denervation

## Abstract

Atherosclerosis is characterized by the narrowing of the arterial lumen due to subendothelial lipid accumulation, with hypercholesterolemia being a major risk factor. Despite the recent advances in effective lipid-lowering therapies, atherosclerosis remains the leading cause of mortality globally, highlighting the need for additional therapeutic strategies. Accumulating evidence suggests that the sympathetic nervous system plays an important role in atherosclerosis. In this article, we reviewed the sympathetic innervation in the vasculature, norepinephrine synthesis and metabolism, sympathetic activity measurement, and common signaling pathways of sympathetic activation. The focus of this paper was to review the effectiveness of pharmacological antagonists or agonists of adrenoceptors (α1, α2, β1, β2, and β3) and renal denervation on atherosclerosis. All five types of adrenoceptors are present in arterial blood vessels. α1 blockers inhibit atherosclerosis but increase the risk of heart failure while α2 agonism may protect against atherosclerosis and newer generations of β blockers and β3 agonists are promising therapies against atherosclerosis; however, new randomized controlled trials are warranted to investigate the effectiveness of these therapies in atherosclerosis inhibition and cardiovascular risk reduction in the future. The role of renal denervation in atherosclerosis inhibition in humans is yet to be established.

## 1. Introduction

Atherosclerosis is an arterial disease that is characterized by the narrowing of the arterial lumen due to the subendothelial accumulation of lipids [1,2,3]. Atherosclerosis is the key underlying mechanism for ischemic heart disease and stroke [4]. Despite the availability of a wide array of effective lipid-lowering medications such as statins, ezetimibe, and PCSK9 (proprotein convertase subtilisin-like kexin type 9) inhibitors [5,6,7,8], ischemic heart disease and stroke remain the leading two causes of mortality globally [9], highlighting the need to identify new therapeutic strategies for atherosclerosis.

Arteries are innervated organs [10], and the possible contribution of the sympathetic nervous system to atherosclerosis has been highlighted recently [11,12]. In this review, we aimed to summarize the effectiveness of pharmacological antagonists or agonists of adrenergic receptors (α1, α2, β1, β2, and β3 adrenoceptors) and renal denervation on atherosclerosis.

## 2. Sympathetic Innervation in the Vasculature

Major arteries and precapillary arterioles are innervated by sympathetic nerves, but other vessels, such as venules, capillaries, and collecting veins, are rarely innervated [13]. The integration of the sympathetic nervous system’s efferent activities to the blood vessels happens in the medulla oblongata of the brainstem, the activities of which are regulated by the hypothalamus and cerebral cortex [14,15].

**Figure 1:**
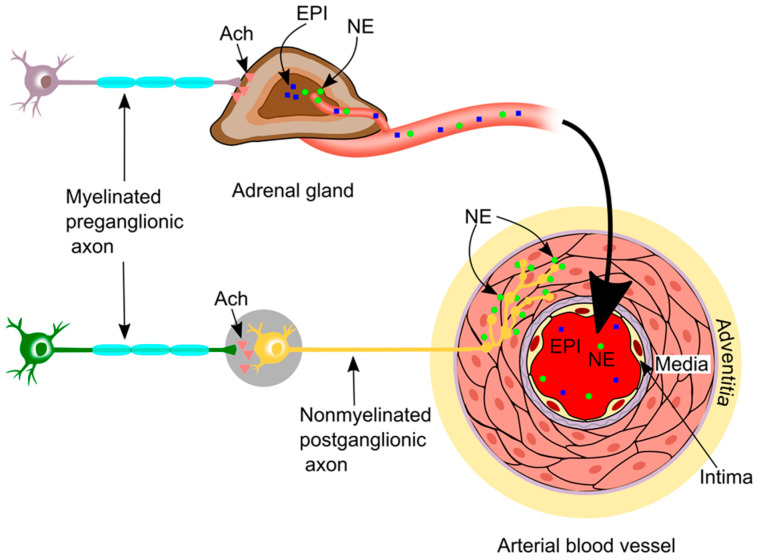
Sympathetic innervation in vasculature. The sympathetic pathway is formed of two serially connected sets of neurons: preganglionic and postganglionic neurons. The preganglionic neurons originate in the brainstem or the spinal cord. They exit the spinal cord and synapse (using acetylcholine as a neurotransmitter) with postganglionic sympathetic neurons in the ganglia. The nerve endings of the postganglionic neurons branch repeatedly, forming synapses en passant (“synapses in passing”) or varicosities (knoblike swellings) containing mitochondria and synaptic vesicles. The key neurotransmitter in the synaptic vesicles of the varicosities is norepinephrine. In addition, the sympathetic preganglionic neurons synapse with chromaffin cells in the adrenal gland to stimulate the production of epinephrine and norepinephrine from the adrenal medulla. The produced epinephrine and norepinephrine then enter the blood and may affect distant blood vessels and tissues. Ach, acetylcholine; EPI, epinephrine; and NE, norepinephrine.

The sympathetic pathway is formed via two serially connected sets of neurons [16,17] (Figure 1). The first (preganglionic) set of neurons originates in the brainstem or the spinal cord. These sympathetic preganglionic neurons are myelinated. They exit the spinal cord from the ventral roots and synapse with the postganglionic sympathetic neurons (second set) in the ganglia, with acetylcholine as the neurotransmitter. Therefore, the preganglionic sympathetic neurons are cholinergic. The postganglionic neuronal axons travel to the vascular smooth muscle, and the nerve endings branch repeatedly, each branch forming synapses en passant “synapses in passing” with vascular smooth muscle cells [16]. Each nerve ending has a series of varicosities (knoblike swellings) containing mitochondria and synaptic vesicles, which make the nerve endings resemble a string of beads, with the beads ~1 µm wide and separated by ~4 µm [18]. The structure of the sympathetic nerve and smooth muscle synapse has three parts: the presynaptic part (varicosities of the nerve ending), the postsynaptic part (the smooth muscle membrane), and the synaptic cleft (the area between the presynaptic and postsynaptic parts) [19]. The neurotransmitter in the synaptic vesicles of the varicosities is norepinephrine, and, therefore, the postganglionic sympathetic neurons are adrenergic. Exceptions to this are the sympathetic postganglionic neurons to blood vessels in the skin; these neurons secrete acetylcholine and therefore are cholinergic [14]. However, blood vessels in the skin will not be covered by this review as they are not thought to be involved in atherosclerosis.

In addition, the sympathetic preganglionic neurons synapse with chromaffin cells in the adrenal gland. As a result, the adrenal medulla produces epinephrine (80%) and norepinephrine (20%) into circulation [20]. These two hormones (rather than neurotransmitters, as they function in the manner of hormones) enter the blood and circulate around the body [20]. Therefore, circulating epinephrine and norepinephrine can affect distant blood vessels [17]. Norepinephrine induces vasoconstriction in the skin and viscera and shifts blood flow to other areas, such as exercising skeletal muscle. Epinephrine induces vasodilation of the blood vessels in skeletal muscle at the onset of the “fight or flight” response [20].

## 3. Norepinephrine Synthesis and Metabolism

**Figure 2:**
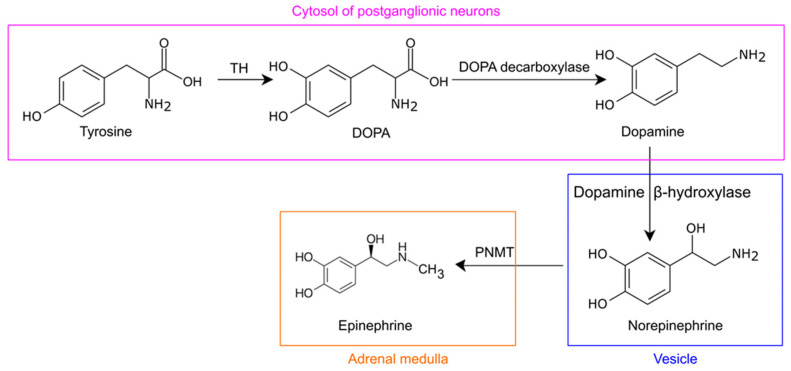
Norepinephrine biosynthesis. Tyrosine is converted by tyrosine hydroxylase (TH) to dihydroxyphenylalanine (DOPA), and the latter is converted by DOPA decarboxylase to dopamine in the cytoplasm. Dopamine is converted by dopamine β-hydroxylase to norepinephrine in the vesicles. In the adrenal medulla, norepinephrine is converted by phenylethanolamine N-methyltransferase (PNMT) to epinephrine.

Norepinephrine is synthesized in the postganglionic neurons from tyrosine, which is synthesized from phenylalanine and can also be obtained directly from food (Figure 2). Tyrosine is converted by tyrosine hydroxylase to dihydroxyphenylalanine (DOPA); DOPA, in turn, is converted by DOPA decarboxylase to dopamine in the cytoplasm. Dopamine is taken up into vesicles and converted by dopamine β-hydroxylase to norepinephrine [21]. In the adrenal medulla, norepinephrine is converted by phenylethanolamine N-methyltransferase to epinephrine [20].

Norepinephrine is stored in vesicles in the nerve terminals, where it is concentrated and protected from metabolism until release upon nerve stimulation. The effects of norepinephrine are terminated mainly through reuptake back into nerve terminals by a high-affinity transporter. Norepinephrine can also be metabolized to inactive products.

Much of the norepinephrine released from adrenergic fibers is removed from the synapse by active transport back into the nerve endings in a process called reuptake [14]. Monoamine oxidase in the mitochondria of the synaptic knob, together with other enzymes, will inactivate norepinephrine [22]. This may take a few seconds, during which some molecules may diffuse into nearby tissues or the bloodstream, where other enzymes decompose them. Some norepinephrine molecules, however, may escape decomposition and remain active for a while. In fact, norepinephrine and epinephrine released from the adrenal medulla upon sympathetic stimulation can exist in the blood for up to 20 min [14].

**Figure 3:**
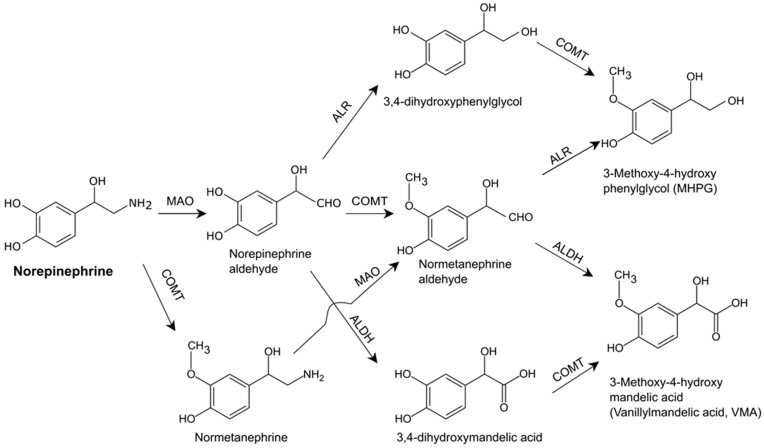
Norepinephrine metabolism. ALDH, aldehyde dehydrogenase; ALR, aldehyde reductase; COMT, catechol-O-methyltransferase; and MAO, monoamine oxidase.

Norepinephrine is metabolized by various enzymes including monoamine oxidase, catechol-O-methyltransferase, aldehyde reductase, and aldehyde dehydrogenase, and the final products are 3-methoxy-4-hydroxyphenylglycol (MHPG) and 3-methoxy-4-hydroxymandelic acid (vanillylmandelic acid) [23,24,25] (Figure 3). The major metabolite found in the blood and urine is MHPG [26,27], and levels of this metabolite are frequently used to assess the functional status of the noradrenergic system in human subjects [28].

## 4. Measurements of Sympathetic Nerve Activity

The methods for measuring the activity of sympathetic nerves can be categorized into those measuring global sympathetic nerve activity and those measuring regional sympathetic nerve activity. Global activity can be measured by assessing the norepinephrine concentration in the blood or urine [29,30,31], and regional activity can be measured by norepinephrine spill over [32,33], clinical microneurography [34,35,36,37,38,39], and sympathetic imaging [40,41,42]. The advantages and disadvantages of these methods are listed in Table 1.

## 5. Adrenergic Receptors (Adrenoceptors) in the Vasculature

There are two types of adrenoceptors: alpha (α) and beta (β). α adrenoceptors include α1 and α2, and β adrenoceptors include β1, β2, and β3. All of these adrenoceptors are G protein-coupled receptors (GPCRs) with seven transmembrane domains [43,44]. They respond to norepinephrine and epinephrine by producing a response within the cell involving a second messenger or ion channel [43]. Although norepinephrine has a stronger effect on α adrenoceptors, it can stimulate both α and β adrenoceptors. Consequently, the way sympathetic activation influences effector cells depends on the relative numbers of α and β adrenoceptors in the cell membrane [14].

All five types of adrenoceptors are expressed in vasculature [45]. In particular, α1 adrenoceptors are predominately expressed in the peripheral arterial blood vessels [46,47,48]; β1 adrenoceptors are distributed in the thoracic aorta, carotid, femoral, and pulmonary arteries; β2 adrenoceptors are distributed in the aorta and carotid arteries [49]; and β3 adrenoceptors are distributed in the blood vessels in the skin [50].

These adrenoceptors are also richly expressed in other organs. For example, α1 adrenoceptors are expressed in the airway and urinary tract [51]; β1 adrenoceptors are richly expressed in the heart, kidney, and fat cells [52]; β2 adrenoceptors are located in the bronchial tract, pancreas, uterus, liver, and endocrine glands [53]; and β3 adrenoceptors are located in the urinary bladder [54], brown adipose tissue, white adipose tissue, and myocardium [55].

## 6. Molecular Pathways Underlying Sympathetic Activation-Induced Vasoconstriction and Relaxation

### 6.1. Sympathetic Activation-Induced Vasoconstriction under Physiological Conditions

**Figure 4:**
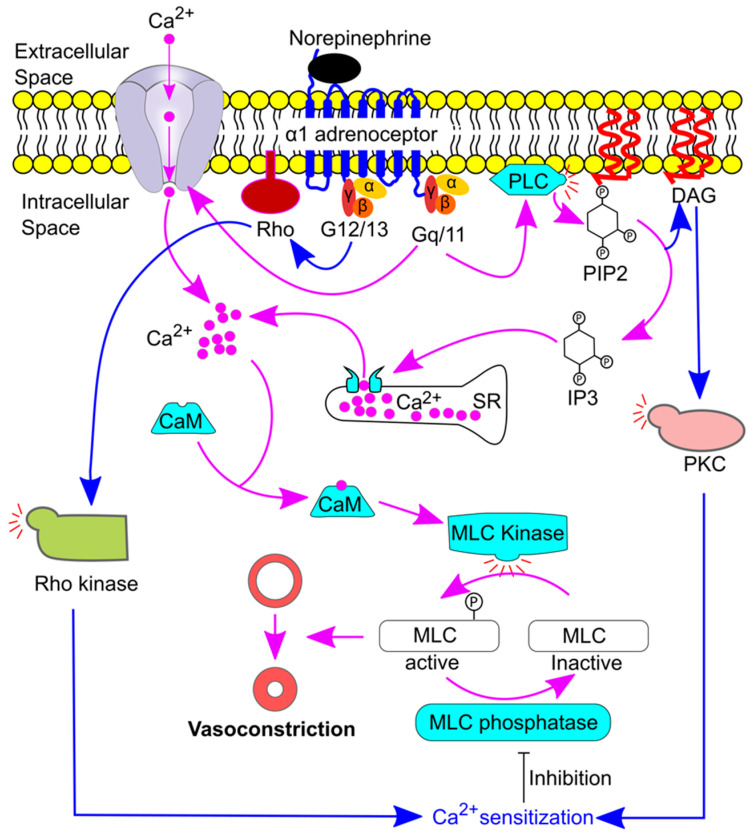
Illustration of norepinephrine-induced vasoconstriction via an α1 adrenoceptor. Norepinephrine binds the α1 adrenoceptor, resulting in Gq/11 activation and opening of the calcium (Ca^2+^) channel. Thus, the cytoplasmic Ca^2+^ concentration increases. In addition, Gq/11 induces inositol triphosphate (IP3) production which binds to IP3 receptors on the sarcoplasmic reticulum (SR), resulting in the release of Ca^2+^ from SR. Free Ca^2+^ then binds to calmodulin (CaM) and phosphorylates the myosin light chain (MLC), which leads to vasoconstriction. In addition, activation of the α1 adrenoceptor can lead to the activation of Rho kinase and protein kinase C (PKC), which results in Ca^2+^ sensitization by phosphorylating and inhibiting MLC phosphatase. DAG, diacylglycerol; P, phosphate; and PLC, phospholipase C.

The basal sympathetic activity plays a pivotal role in maintaining vascular tone, as a sympathetic ganglionic blockade decreases blood pressure [56]. Consistently, sympathetic activation can increase blood pressure, mediated by vasoconstriction via α adrenoceptor activation [46,57]. Activation of α1 adrenoceptors leads to an increase in the calcium (Ca^2+^) concentration in the cytosol of the vascular smooth muscle cells and consequently triggers a contractile response [58] (Figure 4). First, the binding of norepinephrine or epinephrine to the α1 adrenoceptor (G protein-coupled receptor) leads to the opening of Ca^2+^ channels (L-type and T-type) in the cell membrane of the vascular smooth muscle cells [43,46,58], which increases the cytoplasmic Ca^2+^ concentration ([Ca^2+^]_i_). In addition, the G protein Gq/11 activates phospholipase C, which converts phosphatidylinositol 4,5-bisphosphate (PIP2) to diacylglycerol (DAG) and inositol 1,4,5-triphosphate (IP3). IP3 will then bind to the IP3 receptor on the sarcoplasmic reticulum (SR) to release stored Ca^2+^. Once Ca^2+^ enters the cell it will bind and activate calmodulin. Calmodulin then activates myosin light chain (MLC) kinase. Phosphorylation of MLC by MLC kinase leads to a conformational change in the myosin head, which increases myosin ATPase activity and promotes interaction between the myosin head and actin. Cross-bridge cycling then occurs, and tension is generated. Dephosphorylation of MLC by MLC phosphatase terminates smooth muscle contraction [58,59].

[Ca^2+^]_i_ is vital for the smooth muscle to contract. However, the contractility at a given level of [Ca^2+^]_i_ could vary, and under some conditions, the same concentration or even a lower cytoplasmic Ca^2+^ concentration can produce high contractile force. This phenomenon is called Ca^2+^ sensitization [60,61]. In vascular smooth muscle cells, Ca^2+^ sensitization is mediated by protein kinase C (PKC) and Rho kinase via inhibition of MLC phosphatase [60] (Figure 4). DAG, produced by PLC activity, activates PKC which then phosphorylates and inhibits MLC phosphatase [60,62]. In addition, the G12/13 signaling pathway existing in vascular smooth muscle [63] could lead to the activation of Rho and Rho kinase, which then phosphorylates and inhibits MLC phosphatase [62].

### 6.2. Activation of β Adrenoceptors Induces Vasorelaxation

Activation of β adrenoceptors by isoproterenol induces vasorelaxation in the thoracic aorta, carotid, femoral, and pulmonary arteries [49,50,64], suggesting that β adrenoceptors play a modulatory role in vascular tone regulation. Activation of β adrenoceptors (GPCR) leads to Gs activation, which activates adenylyl cyclase to increase cyclic adenosine monophosphate (cAMP) [65]. cAMP then activates protein kinase G (PKG), which opens the large-conductance, Ca^2+^-activated potassium (BK_Ca_) channel, thus relaxing the vascular smooth muscle [66]. In large pulmonary arteries, the activation of β adrenoceptors can also increase nitric oxide production by the endothelial cells [67], which leads to vasorelaxation.

## 7. Roles of Adrenoceptors in Atherosclerosis

### 7.1. Role of α1 Adrenoceptors in Atherosclerosis

α1 blockers have been frequently shown to inhibit atherosclerosis in research animals [68,69,70] and humans [71], although certain studies fail to demonstrate a beneficial effect of α1 blockers [72]. The anti-atherosclerotic effects of α1 blockers may be mediated by their blood pressure-lowering effect and their favorable effect on lipid profile [68,71,72,73]. It has been shown that α1 blockers decrease plasma total cholesterol, very low-density lipoprotein (VLDL), and triglycerides [68,71,72,73], and increase high-density lipoprotein (HDL) cholesterol [71]. Despite these beneficial effects, α1 blockers may increase the risk of heart failure in the long term [74]. The mechanism underlying this side effect is unknown. As α1 adrenoceptors are also expressed in cardiomyocytes [47], it is possible that α1 blockers modify gene expression in cardiomyocytes via the α1 adrenoceptor-stimulatory Gs protein pathway and consequently weaken the structure or function of the heart over a long period of time.

### 7.2. Role of α2 Adrenoceptors in Atherosclerosis

α2 activation leads to activation of the inhibitory Gi protein which inhibits adenylate cyclase and decreases intracellular cAMP [51]. In addition, α2 activation can lead to a decrease in cytoplasmic Ca^2+^, which then leads to a decrease in neurotransmitter release [75]. Both central and peripheral activation of α2 adrenoceptors can reduce sympathetic activity [51].

**Figure 5:**
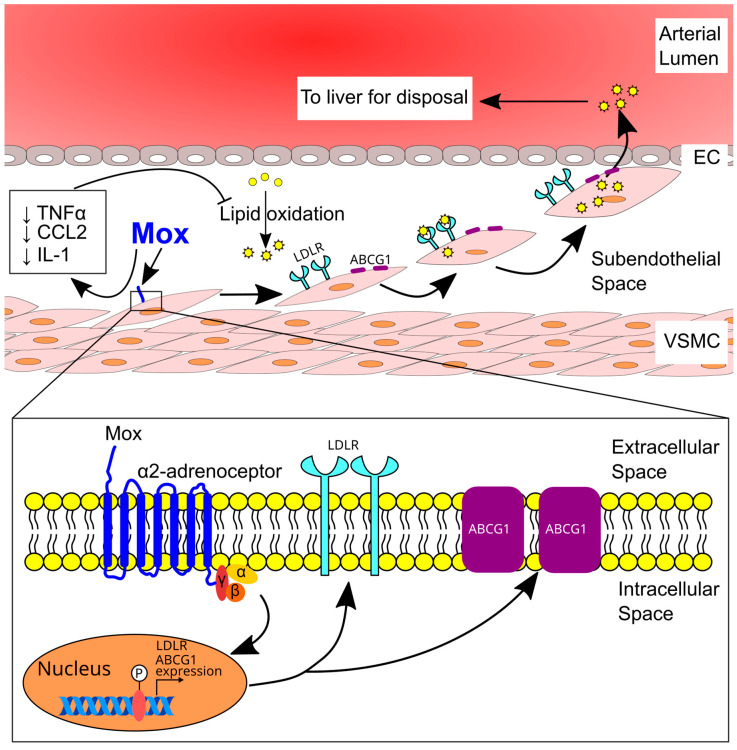
Moxonidine-induced inhibition of atherosclerosis. Moxonidine decreases the expression of inflammatory genes, inhibits the oxidation of LDL, and enhances VSMC migration. VSMCs then migrate to a location that could facilitate both oxidized LDL uptake via the LDL receptor and its efflux back to circulation via the ABCG1 transporter for detoxification by the liver. ↓, decrease; ABCG1, ATP binding cassette subfamily G member 1; CCL2, chemokine ligand 2 (also known as monocyte chemoattractant protein-1); EC, endothelial cell; IL, interleukin; LDL, low-density lipoprotein; LDLR, low-density lipoprotein receptor; Mox, moxonidine; TNF-α, tumor necrosis factor-α; and VSMC, vascular smooth muscle cell. This figure is from Wang et al. with permission [76].

The involvement of α2 adrenoceptors in atherosclerosis is not clear. A recent study has shown that moxonidine, an agonist for α2 and imidazoline 1 (I1) receptors, inhibits atherosclerosis in apolipoprotein E-deficient (ApoE^−/−^) mice [76], possibly via inhibiting inflammation and promoting oxidized LDL uptake and clearance (Figure 5). This anti-atherosclerotic effect seems to be mediated by α2 adrenoceptors as the effect of moxonidine on oxidized LDL uptake by cultured vascular smooth muscle cells was inhibited by the α2 antagonist RX821002; in addition, activation of I1 by AGN192403 did not replicate the effects of moxonidine on oxidized LDL uptake. The role of α2 adrenoceptors in atherosclerosis needs to be investigated in the future using specific α2-agonists.

### 7.3. Role of β1 Adrenoceptors in Atherosclerosis

Many preclinical [77,78,79,80,81,82,83] and clinical studies [84,85] have been conducted in recent years to investigate the effect of β blockers on atherosclerosis (Table 2). It has been shown that the first generation of β blockers (non-selective β1/β2 blockers) [77], the second generation of β blockers (selective β1 blockers) [78,79,84,85], and the third generation of β blockers (β blockers with additional properties) [80,81,82,83] attenuate atherosclerosis progression. In particular, two randomized controlled trials showed that the selective β1 blocker metoprolol reduced the rate of atherosclerosis progression [84,85], even in the presence of lipid-lowering therapy [85]. In these two trials, metoprolol was used at a low dose of 25–100 mg daily in a controlled-release/extended-release formulation [84,85].

β blockers inhibit atherosclerosis via multiple mechanisms including inhibiting inflammation (Table 2). For example, the β1-selective blocker metoprolol decreases the circulating level of proinflammatory cytokines and chemokines and decreases the macrophage content in the lesion [79]. Similarly, the non-selective inhibition of β1 and β2 adrenoceptors by propranolol decreases the production of granulocyte-macrophage progenitors (GMPs) in the bone marrow and decreases the circulating numbers of monocytes and neutrophils [77]. The third generation of β blockers has other anti-atherosclerotic mechanisms, including promoting cholesterol efflux [80], inhibiting oxidative stress [81], preventing LDL oxidation [82], improving endothelial function [82,83], and decreasing monocyte adhesion to endothelium [83].

It is worth noting that some studies have shown that β blockers (non-selective β1/β2 blockers or selective β1 blockers) have adverse effects on plasma lipoprotein levels such as increasing VLDL and decreasing HDL cholesterol, and, therefore, β blockers are currently discouraged for use to prevent atherosclerosis [53]. However, recent studies have reported that β blockers do not affect plasma levels of total cholesterol, triglycerides, or HDL cholesterol [79,81,83,86].

In addition, the relationship between the baseline use of β blockers and clinal outcomes in epidemiological studies is inconsistent. For example, it has been reported that baseline β1 blocker use was associated with lower 30-day and 10-year mortality in 3371 patients undergoing major vascular surgery [87]. However, it has also been reported that baseline β blocker use was associated with an increased cardiovascular event risk in 11,785 patients undergoing infrainguinal revascularization for critical limb ischemia [88] and in 14,671 patients with type 2 diabetes and established atherosclerotic cardiovascular disease [89]. Moreover, Cimaglia et al. found that baseline β blocker use was not associated with an increased cardiovascular event risk in 618 diabetic patients with the most advanced stage of peripheral artery disease [90]. Therefore, more randomized controlled trials are needed to establish the merit of the use of β blockers for atherosclerosis inhibition and cardiovascular risk reduction.

### 7.4. Role of β2 Adrenoceptors in Atherosclerosis

Whether β2 adrenoceptors play a role in atherosclerosis is unknown and this is worthy of investigation in the future. It has been shown that the selective blockade of β2 adrenoceptors could reduce inflammation and oxidative stress [91], suggesting that β2 adrenoceptors may be involved in atherosclerosis.

### 7.5. Role of β3 Adrenoceptors in Atherosclerosis

#### 7.5.1. Preclinical Studies

β3 agonists inhibit atherosclerosis in mice fed with a Western-type diet in preclinical studies [92,93,94] (Table 3). They inhibit atherosclerosis via multiple mechanisms, e.g., (1) activating brown adipose tissue and thus increasing fat oxidation and decreasing body fat mass, (2) increasing the clearance of plasm triglyceride-rich lipoprotein (TRL), i.e., VLDL and chylomicrons, via increasing the liver uptake of VLDL core remnants and thus decreasing plasma non-HDL cholesterol, (3) increasing HDL cholesterol via promoting the transfer of TRL particles to HDL particles, (4) increasing lipoprotein lipase lipolysis activity and thus decreasing plasma triglycerides, and (5) decreasing total cholesterol [92,93,94,95]. The resultant favorable lipid profile may explain the anti-atherosclerotic effect of β3 agonism.

It is worth noting that β3 agonism does not affect atherosclerosis severity [94], lesion composition (smooth muscle cells, collagen, and macrophages), nor the stability of the lesion as assessed by the ratio of stable markers (collagen area or combined collagen and smooth muscle cell area) versus the unstable markers (macrophage area) [92,93].

#### 7.5.2. Clinical Studies

β3 adrenoceptors are expressed in a species-specific manner. For example, in mice, they are most highly expressed in white and brown adipose tissues, whereas in humans they are most highly expressed in the urinary bladder [54]. A β3 agonist mirabegron (Myrbetriq, extended-release tablet, Astellas Pharma) has been approved for the treatment of overactive bladder, with an approved maximum dose of 50 mg per day.

β3 agonism by mirabegron increases brown adipose tissue volume, brown adipose tissue metabolic activity, lipolysis, fat oxidation, and resting energy expenditure in humans [54,96,97]. It also increases HDL cholesterol, apolipoprotein A1 (ApoA1), and ApoE [96]. These results suggest that β3 agonism may inhibit atherosclerosis in humans.

It is worth noting that high doses of mirabegron have unfavorable cardiovascular effects. Mirabegron increased the heart rate, blood pressure, and rate-pressure product (an indicator of myocardial oxygen consumption) in clinical trials at doses higher than the approved maximum therapeutic dose of 50 mg per day [54,96,97,98]. In addition, mirabegron caused QT prolongation in women but not men at the supratherapeutic dose of 200 mg per day [98]. These cardiovascular side effects were not significant at the therapeutic 50 mg dose [54,98]. The reason for the high-dose mirabegron-induced cardiovascular stimulation is unknown. It is possible that the high dose of mirabegron is taken up by sympathetic nerve terminals which causes the release of norepinephrine and activation of β1 adrenoceptors in cardiomyocytes [96]. Therefore, a low dose of mirabegron should be used in future clinical trials investigating the potential anti-atherosclerotic effect of β3 agonism.

## 8. Renal Denervation and Atherosclerosis

Renal denervation is used to lower the blood pressure of hypertensive patients via the inhibition of sympathetic activity [99,100,101]. It may have beneficial effects in other indications beyond hypertension, such as renal failure [102,103] and atrial fibrillation [104]. Here, we summarize the recent studies investigating the effect of renal denervation on atherosclerosis in preclinical and clinical studies.

### 8.1. Preclinical Studies

**Figure 6:**
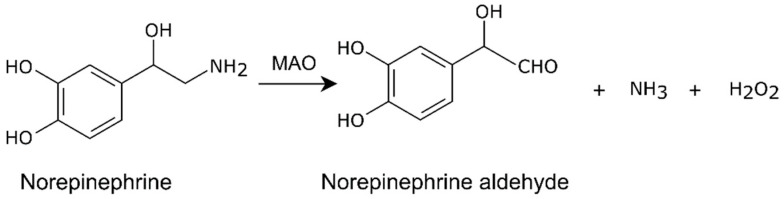
Production of oxidants by norepinephrine metabolism.

Three preclinical studies have shown that renal denervation decreased atherosclerosis independent of blood pressure in ApoE^−/−^mice fed with a high-fat diet [105,106,107]. This may be mediated by a decrease in monoamine oxidase (MAO). Norepinephrine is metabolized by MAO to produce oxidants including aldehyde and hydrogen peroxide (Figure 6). High-fat diet feeding in mice increases MAO activity [105,108], which impairs mitochondrial homeostasis and increases reactive oxygen species (ROS) production and nuclear factor kappa B (NF-κB) activation [105]. Renal denervation decreased aortic MAO-A and inflammation, and decreased macrophage accumulation in the lesion [105]. The anti-inflammatory effect of renal denervation in high-fat diet-fed mice has been confirmed by another two studies [106,107] (Table 4).

In contrast, two preclinical studies found that renal denervation increased atherosclerosis in mice [109] and minipigs [110] (Table 4). The pro-atherosclerotic effect of renal denervation is associated with an increase in matrix metalloproteinase-2 (MMP-2, a pro-atherosclerotic marker) [109,111,112,113] and endothelin-1 [110].

Activated MMP-2 is an alternative to the endothelin converting enzyme as it can convert the big inactive precursor big endothelin-1 to active endothelin-1 [114,115]. Endothelin activates NF-κB in human endothelial cells [116] and increases NADPH oxidase and superoxide production in pulmonary arteries [117]. Renal denervation increased MMP-2 [109] and endothelin-1 protein and its receptors [110], which was accompanied by the activation of NF-κB and NADPH oxidase pathways [110]. The renal denervation-induced increase in oxidative stress was indicated by an increase in 4-hydroxynonenal [110], a marker of oxidative stress and atherosclerosis [118]. In addition, renal denervation may inhibit the endothelial nitric oxide synthase–nitric oxide (eNOS-NO) pathway which may promote atherosclerosis [110].

### 8.2. Clinical Studies

It has been shown that renal denervation in humans, a blood-pressure-lowering therapy [119,120,121], might slightly increase the occurrence of renal artery stenosis, with an occurrence rate of 0.3–2.2%, although some studies reported a higher rate of more than 10% [122,123].

In a clinical study with a small sample size (*n* = 39), renal denervation did not affect atherosclerosis after 12 months in those with resistant hypertension (Table 4). However, the longer-term effect of renal denervation on atherosclerosis in humans is not available and needs to be investigated in the future. Careful consideration should be given when performing renal denervation in patients with severe atherosclerosis or stenosis [124,125,126,127,128]. It has been reported that renal denervation increases MMP-2 in both animals [109] and humans [129], and an increase in MMP-2 may adversely affect the stability of severe atherosclerosis [130].

**Table 4:** Effect of renal denervation on atherosclerosis in preclinical and clinical studies.

Patients/Animals	Effect on Atherosclerosis	Mechanisms	Reference
Preclinical Studies			
ApoE^−/−^mice HFD for 20 weeks	↓	↓ MAO-A ↓ CCL2, ICAM-1 ↓ Macrophage ↓ ROS ↓ NF-κB	[105]
ApoE^−/−^mice HFD for 6–12 weeks	↓	↓ TNFα, IL-Iβ, etc ↓ Circulating neutrophils ↓ Circulating monocytes	[106]
ApoE^−/−^mice HFD for 10 weeks	↓	↑ VSMC ↓ CCL2 and 8-isoprostane	[107]
ApoE^−/−^mice Angiotensin II fusion	↑	↑ MMP-2	[109]
Minipigs HFD for 6 months	↑	↑ ET-1 ↑ ET-1 A and Breceptors ↑ NOX2 ↑ NF-κB ↑ 4-hydroxynonenal ↓ eNOS phosphorylation ↓ NO	[110]
Clinical studies			
39 patients with rHTN	↔	NR	[131]

↔, no effect; ↑, increase; ↓, decrease; ApoE^−/−^, apolipoprotein E-deficient; CCL2, chemokine ligand 2; eNOS, endothelial nitric oxide synthase; ET-1, endothelin-1; HFD, high fat diet; ICAM, intercellular adhesion molecule; IL, interleukin; MAO, monoamine oxidase; MMP-2, matrix metalloproteinase-2; NF-κB, nuclear factor kappa B; NO, nitric oxide; NOX, NADPH oxidase; NR, not reported; rHTN, resistant hypertension; ROS, reactive oxygen species; TNFα, tumor necrosis factor α; and VSMCs, vascular smooth muscle cells.

## 9. Artery–Brain Circuit and Atherosclerosis

### 9.1. Establishment of the Artery–Brain Circuit in Mice

A significant development in vascular sympathetic innervation is the recent establishment of the artery–brain circuit in the mouse abdominal aorta by Mohanta et al. [11]. In the artery–brain circuit sensor pathway, signals from the sensory afferent neurons, which innervate the abdominal aorta, pass through the dorsal root ganglia, enter the spinal cord via the spinal cord dorsal horn, and then transmit to the brain stem medulla oblongata. In the artery–brain circuit effector pathway, sympathetic signals from the hypothalamic and brainstem nuclei pass the spinal cord and then the coeliac ganglia and finally reach the aortic adventitia; in addition, parasympathetic signals from the medulla oblongata can pass on to the coeliac ganglia via the vagal nerve [11].

The artery–brain circuit plays an important role in regulating vascular inflammation. It has been shown that coeliac ganglionectomy, which was confirmed by a decrease in norepinephrine content and the density of tyrosine hydroxylase-positive sympathetic nerves in the aorta, decreased the number and size of artery tertiary lymphoid organs (ATLOs, structures in the adventitia where infiltrated immune cells aggregate), and attenuated T and B cell vascular infiltration in aged ApoE^−/−^mice [11].

### 9.2. Ganglionectomy and Atherosclerosis

Disruption of the artery–brain circuit by coeliac ganglionectomy decreased the atherosclerotic plaque size in the aortic arch and abdominal aorta of aged ApoE^−/−^mice as assessed by ultrasound imaging [11]. The anti-atherosclerotic effect of coeliac ganglionectomy was associated with the decreased vascular infiltration of immune cells [11]. These results suggest that the artery–brain circuit may promote vascular inflammation and atherosclerosis.

It is worth noting that contradicting results exist. A study by Murphy et al., conducted in the 1950s, showed that removal of L1 to L4 ganglia increased atherosclerosis in the abdominal aorta and femoral arteries of rabbits fed a high-cholesterol diet [132]. To assure adequate denervation, Murphy et al. performed “a peri-aortic stripping of all adventitia” of the infrarenal aorta in addition to the removal of the ganglia. Whether this additional procedure explains the observed inconsistency needs to be investigated in the future.

## 10. Sympathetic Nervous System and Peripheral Artery Disease (PAD)

PAD, also known as atherosclerotic occlusive disease of the lower extremities, affects more than 200 million people worldwide [133]. Patients with PAD display cardiac autonomic dysfunction [134], increased sympathetic nerve activity, and an augmented blood pressure response to exercise [135,136]. The increase in sympathetic nerve activity in PAD is associated with an increase in sensory nerve receptors (e.g., receptor potential vanilloid type 1, purinergic P2X purinoceptor 3, and acid-sensing ion channel subtype 3) [137]. Cardiac autonomic dysfunction has also been shown to predict a higher risk for cardiovascular disease events in atherosclerosis-prone diabetic patients [138,139]. Whether the sympathetic nervous system plays a role in the progression of atherosclerosis underlying PAD needs to be investigated in the future.

## 11. Concluding Remarks

Major arteries and arterioles are innervated by sympathetic nerves and all types of adrenoceptors (α1, α2, β1, β2, and β3) are expressed in arterial blood vessels. α1 blockers inhibit atherosclerosis; however, their use increases the risk of heart failure. Therefore, whether α1 blockers provide a long-term cardiovascular benefit needs to be investigated in the future. α2 stimulation inhibits sympathetic activity and the non-specific α2 agonist moxonidine inhibits atherosclerosis in ApoE^−/−^mice. The definitive role of the α2 adrenoceptors in atherosclerosis needs to be investigated using selective α2 agonists and confirmed in humans.

β blockers (selective and non-selective β1 blockers) are currently not recommended for treating atherosclerosis due to adverse effects on lipoprotein levels, such as increasing VLDL and decreasing HDL cholesterol. However, many recent preclinical studies have shown that β blockers can attenuate atherosclerosis progression without adverse effects on plasma total cholesterol, triglycerides, or HDL cholesterol. Two randomized controlled trials have shown that β blockers reduce the progression of atherosclerosis in humans even in the presence of concomitant lipid-lowering therapy. In addition, many newer generations of β blockers have additional favorable properties, including promoting cholesterol efflux, inhibiting oxidative stress, and improving endothelial function. Therefore, more randomized controlled trials are needed to establish the use of β blockers for atherosclerosis inhibition and cardiovascular risk reduction. In future trials, low-dose and controlled-release formulations of β blockers and newer generations of β blockers are worthy of consideration.

β3 agonism is gaining interest as a potential strategy to inhibit atherosclerosis. Preclinical studies show consistent results on the anti-atherosclerosis effect of β3 agonism, which is mediated by activating brown adipose tissue metabolism and increasing fat oxidation. However, the effect of β3 agonism on atherosclerosis in humans has not been investigated. Given that higher doses of β3 agonists could increase blood pressure and heart rate and increase the risk of QT prolongation, it is worthy of consideration to use low doses of β3 agonists or controlled release formations in future clinical trials.

The effects of renal denervation on atherosclerosis are inconsistent in preclinical studies. Whether renal denervation inhibits atherosclerosis in humans needs to be established in the future.

## Figures and Tables

**Table 1:** Methods for measuring sympathetic nerve activity.

	NE in Blood/Urine	NE Spillover	Clinical Microneurography	Sympathetic Imaging
Global/regional measurement	Global	Regional	Regional	Regional
Advantages	Convenient Little or no invasiveness	Measures the regional rate of NE spillover from the heart or kidneys	Is the only method for the direct measurement of adrenergic activity in humans Precisely assesses resting sympathetic activity and tracks the changes in cardiovascular regulation in response to stimuli	Demonstrates the anatomy of sympathetic innervation of an organ
Disadvantages	Lacks information on regional sympathetic responses	Is highly invasive Requires catheterization of veins draining internal organs	Requires a high degree of skill Requires several months of training	Is unable to differentiate the relative contribution of denervation and dysinnervation
References	[29,30,31]	[32,33]	[34,35,36,37,38,39]	[40,41,42]

NE, norepinephrine.

**Table 2:** Effect of β blockers (β1/β2 blockers) on atherosclerosis in recent studies.

β Blockers	Patients/Animals	Effect on Atherosclerosis	Mechanism	Reference
First Generation: Non-Selective β1 and β2 Blockers				
Propranolol	BPH/ApoE^−/−^mice	↓	↓ HSPC proliferation in the BM ↓ GMPs in the BM ↓ Blood monocytes and neutrophils ↓ Macrophages in the lesion	[77]
**Second Generation: β1-Selective Blockers**				
Metoprolol	ApoE^−/−^mice	↓	N/R	[78]
	ApoE^−/−^mice	↓	↓ Circulating TNFα, CXCL1 ↓ Macrophages in the lesion	[79]
	Subjects without symptoms	↓	N/R	[84]
	Patients with hypercholesterolemia	↓	N/R	[85]
Third Generation: Non-Selective β Blockers with Additional Properties				
Carvedilol With α1- blocking and antioxidant properties	Ldlr^−/−^mice	↓	↑ ABCA1 in exosomes ↑ Cholesterol efflux ↓ Macrophages in the lesion	[80]
	ApoE^−/−^mice	↓	↓ Superoxide production ↓ Macrophage and T cell infiltration	[81]
	Rabbits	↔	↓ LDL oxidation ↑ eNOS expression ↑ Endothelium-dependent relaxation	[82]
Nipradilol With NO-releasing properties	Rabbits	↓	↑ eNOS ↑ Endothelium-dependent relaxation ↓ Monocyte adhesion to EC ↓ Monocyte/macrophage infiltration	[83]
Third Generation: β1-Selective Blockers with Additional Properties				
Nebivolol With NO-releasing property	Rabbits	↓	↓ LDL oxidation ↓ Inflammatory markers ↑ eNOS expression ↑ Endothelium-dependent relaxation	[82]

↓, decrease; ↑, increase; ↔, no effect; ABCA1, ATP binding cassette subfamily A member 1; ApoE^−/−^, apolipoprotein E-deficient; BM, bone marrow; BPH, Schlager hypertensive (blood pressure high) mice; CXCL1, CXC motif chemokine ligand 1; EC, endothelial cells; eNOS, endothelial nitric oxide synthase; GMPs, granulocyte-macrophage progenitors; HSPC, hematopoietic stem and progenitor cells; LDL, low-density lipoprotein; Ldlr^−/−^, LDL receptor-deficient; NO, nitric oxide; NR, not reported; and TNFα, tumornecrosis factor α.

**Table 3:** Effect of β3 agonism on atherosclerosis in preclinical studies.

β3 Agonist	Animals	Effect on Atherosclerosis	Mechanisms	Reference
CL316,243	E3L.CETP mice	↓	↑ Energy expenditure ↑ Fat oxidation by activated BAT ↓ Total body fat mass ↓ Lipid droplet content in BAT ↓ Plasma TG, TC, and non-HDL cholesterol ↑ Plasma TRL clearance ↑ Hepatic cholesterol content ↑ HDL cholesterol	[92]
CL316,243	E3L.CETP mice	↓	↓ TC and TG ↑ VLDL clearance ↑ Liver uptake of VLDL core remnants ↑ Lipoprotein lipase lipolysis activity ↑ Transfer of VLDL to HDL cholesterol ↑ Plasma HDL cholesterol	[93]
CL316,243	E3L.CETP mice	↓	↓ Total fat mass ↓ Plasma TG and non-HDL cholesterol ↑ Plasma clearance and hepatic uptake of cholesterol-enriched TRL remnants. ↑ HDL cholesterol	[94]
CL316,243	E3L.CETP mice	NR	↓ Body fat masss and gonadal WAT ↓ Plasma TG, TC, and non-HDL cholesterol ↑ Clearance of TRL-like particles ↑ Hepatic uptake of TRL-like remnants ↑ Tranfer of TRL particles to HDL particles ↑ Plasma HDL cholesterol	[95]

↓, decrease; ↑, increase; BAT, brown adipose tissue; E3L.CETP mice, APOE*3-Leiden crossing human cholesteryl ester transfer protein mice; HDL, high-density lipoprotein; NR, not reported; TC, total cholesterol; TG, triglyceride; TRL, triglyceride-rich lipoprotein, i.e., very-low-density lipoproteins and chylomicrons; VLDL, very-low-density lipoprotein; and WAT, white adipose tissue.

## Data Availability

Not applicable.

## References

[1] Jebari-Benslaiman S., Galicia-García U., Larrea-Sebal A., Olaetxea J.R., Alloza I., Vandenbroeck K., Benito-Vicente A., Martín C. (2022). Pathophysiology of Atherosclerosis. Int. J. Mol. Sci..

[2] Ouyang Z., Zhong J., Shen J., Zeng Y. (2023). The cell origins of foam cell and lipid metabolism regulated by mechanical stress in atherosclerosis. Front. Physiol..

[3] Shen Y., Ward N.C., Hodgson J.M., Puddey I.B., Wang Y., Zhang D., Maghzal G.J., Stocker R., Croft K.D. (2013). Dietary quercetin attenuates oxidant-induced endothelial dysfunction and atherosclerosis in apolipoprotein E knockout mice fed a high-fat diet: A critical role for heme oxygenase-1. Free Radic. Biol. Med..

[4] Rocha V.Z., Rached F.H., Miname M.H. (2023). Insights into the Role of Inflammation in the Management of Atherosclerosis. J. Inflamm. Res..

[5] Watts G.F., Gidding S.S., Hegele R.A., Raal F.J., Sturm A.C., Jones L.K., Sarkies M.N., Al-Rasadi K., Blom D.J., Daccord M. (2023). International Atherosclerosis Society guidance for implementing best practice in the care of familial hypercholesterolaemia. Nat. Rev. Cardiol..

[6] Poznyak A.V., Sukhorukov V.N., Eremin I.I., Nadelyaeva I.I., Gutyrchik N.A., Orekhov A.N. (2023). Proprotein Convertase Subtilisin/Kexin 9 as a Modifier of Lipid Metabolism in Atherosclerosis. Biomedicines.

[7] Qiao Y.N., Zou Y.L., Guo S.D. (2022). Low-density lipoprotein particles in atherosclerosis. Front. Physiol..

[8] Cholesterol Treatment Trialists Collaboration (2019). Efficacy and safety of statin therapy in older people: A meta-analysis of individual participant data from 28 randomised controlled trials. Lancet.

[9] World Health Organisation (2020). The Top 10 Causes of Death. https://www.who.int/news-room/fact-sheets/detail/the-top-10-causes-of-death.

[10] Santosa S.M., Guo K., Yamakawa M., Ivakhnitskaia E., Chawla N., Nguyen T., Han K.Y., Ema M., Rosenblatt M.I., Chang J.H. (2020). Simultaneous fluorescence imaging of distinct nerve and blood vessel patterns in dual Thy1-YFP and Flt1-DsRed transgenic mice. Angiogenesis.

[11] Mohanta S.K., Peng L., Li Y., Lu S., Sun T., Carnevale L., Perrotta M., Ma Z., Förstera B., Stanic K. (2022). Neuroimmune cardiovascular interfaces control atherosclerosis. Nature.

[12] Mohanta S.K., Weber C., Yin C., Habenicht A.J.R. (2022). The dawn has come for new therapeutics to treat atherosclerosis: Targeting neuroimmune cardiovascular interfaces in artery brain circuits. Clin. Transl. Med..

[13] Hill C.E., Phillips J.K., Sandow S.L. (2001). Heterogeneous control of blood flow amongst different vascular beds. Med. Res. Rev..

[14] Shier D., Butler J., Lewis R. (2018). Nervous System II: Divisions of the Nervous System. Holes Hum. Anat. Physiol..

[15] Baizer J.S., Webster C.J., Witelson S.F. (2022). Individual variability in the size and organization of the human arcuate nucleus of the medulla. Brain Struct. Funct..

[16] Marieb E.N., Hoehn K. (2018). The Peripheral Nervous System and Reflex Activity. Hum. Anat. Physiol..

[17] Marieb E.N., Hoehn K. (2018). The autonomic nervous system. Hum. Anat. Physiol..

[18] Bennett M.R. (1998). Transmission at Sympathetic Varicosities. Physiology.

[19] Jimsheleishvili S., Marwaha K., Sherman A.L. (2022). Physiology, Neuromuscular Transmission. StatPearls [Internet].

[20] Shier D., Butler J., Lewis R. (2018). Endocrine System. Hole’s Hum. Anat. Physiol..

[21] Dey S.K., Saini M., Prabhakar P., Kundu S. (2020). Dopamine β hydroxylase as a potential drug target to combat hypertension. Expert. Opin. Investig. Drugs.

[22] Shier D., Butler J., Lewis R. (2018). Nervous System I: Basic Structure and Function. Hole’s Hum. Anat. Physiol..

[23] Chaudhary A., Kumar P., Rai V. (2021). Catechol-O-methyltransferase (COMT) Val158Met Polymorphism and Susceptibility to Alcohol Dependence. Indian. J. Clin. Biochem..

[24] Jones D.N., Raghanti M.A. (2021). The role of monoamine oxidase enzymes in the pathophysiology of neurological disorders. J. Chem. Neuroanat..

[25] Haggstrom M. (2014). Medical gallery of mikael haggstrom 2014. WikiJournal Med..

[26] Padala N.S.P., Ajjala D.R., Boggavarapu R.K., Pantangi H.R., Thentu J.B., Mohammed A.R., Nirogi R. (2019). LC-MS/MS method for quantification of 3,4-dihydroxyphenylglycol, a norepinephrine metabolite in plasma and brain regions. Bioanalysis.

[27] Raffel D.M., Crawford T.C., Jung Y.W., Koeppe R.A., Gu G., Rothley J., Frey K.A. (2022). Quantifying cardiac sympathetic denervation: First studies of (18)F-fluorohydroxyphenethylguanidines in cardiomyopathy patients. Eur. J. Nucl. Med. Mol. Imaging.

[28] Bylund D.B., Bylund K.C., Daroff R.B., Aminoff M.J. (2014). Norepinephrine. Encyclopedia of the Neurological Sciences.

[29] Grassi G., Quarti-Trevano F., Esler M.D. (2021). Sympathetic activation in congestive heart failure: An updated overview. Heart Fail. Rev..

[30] Dobrek L. (2021). An Outline of Renal Artery Stenosis Pathophysiology-A Narrative Review. Life.

[31] Esler M., Lambert G., Brunner-La Rocca H.P., Vaddadi G., Kaye D. (2003). Sympathetic nerve activity and neurotransmitter release in humans: Translation from pathophysiology into clinical practice. Acta Physiol. Scand..

[32] Esler M., Jennings G., Lambert G., Meredith I., Horne M., Eisenhofer G. (1990). Overflow of catecholamine neurotransmitters to the circulation: Source, fate, and functions. Physiol. Rev..

[33] Esler M. (2011). The sympathetic nervous system through the ages: From Thomas Willis to resistant hypertension. Exp. Physiol..

[34] Grassi G., Seravalle G., Arenare F., Buccianti G., Furiani S., Ilardo V., Bolla G., Mancia G. (2009). Behaviour of regional adrenergic outflow in mild-to-moderate renal failure. J. Hypertens..

[35] Grassi G., Seravalle G., Dell’Oro R., Arenare F., Facchetti R., Mancia G. (2009). Reproducibility patterns of plasma norepinephrine and muscle sympathetic nerve traffic in human obesity. Nutr. Metab. Cardiovasc. Dis..

[36] Esler M. (2022). Pivotal role of the sympathetic nerves of the human heart in mental stress responses and triggered cardiovascular catastrophes. Auton. Neurosci..

[37] Lambert E., Dawood T., Schlaich M., Straznicky N., Esler M., Lambert G. (2008). Single-unit sympathetic discharge pattern in pathological conditions associated with elevated cardiovascular risk. Clin. Exp. Pharmacol. Physiol..

[38] Seravalle G., Dimitriadis K., Dell’Oro R., Grassi G. (2013). How to assess sympathetic nervous system activity in clinical practice. Curr. Clin. Pharmacol..

[39] Vallbo A.B., Hagbarth K.E., Wallin B.G. (2004). Microneurography: How the technique developed and its role in the investigation of the sympathetic nervous system. J Appl Physiol (1985).

[40] Zelt J.G.E., deKemp R.A., Rotstein B.H., Nair G.M., Narula J., Ahmadi A., Beanlands R.S., Mielniczuk L.M. (2020). Nuclear Imaging of the Cardiac Sympathetic Nervous System: A Disease-Specific Interpretation in Heart Failure. JACC Cardiovasc. Imaging.

[41] Grkovski M., Zanzonico P.B., Modak S., Humm J.L., Narula J., Pandit-Taskar N. (2022). F-18 meta-fluorobenzylguanidine PET imaging of myocardial sympathetic innervation. J. Nucl. Cardiol..

[42] Turnock S., Turton D.R., Martins C.D., Chesler L., Wilson T.C., Gouverneur V., Smith G., Kramer-Marek G. (2020). (18)F-meta-fluorobenzylguanidine ((18)F-mFBG) to monitor changes in norepinephrine transporter expression in response to therapeutic intervention in neuroblastoma models. Sci. Rep..

[43] Docherty J.R. (2010). Subtypes of functional alpha1-adrenoceptor. Cell Mol. Life Sci..

[44] Sheng Y., Zhu L. (2018). The crosstalk between autonomic nervous system and blood vessels. Int. J. Physiol. Pathophysiol. Pharmacol..

[45] Manzini S., Pinna C., Busnelli M., Cinquanta P., Rigamonti E., Ganzetti G.S., Dellera F., Sala A., Calabresi L., Franceschini G. (2015). Beta2-adrenergic activity modulates vascular tone regulation in lecithin:cholesterol acyltransferase knockout mice. Vascul Pharmacol..

[46] Chen Z.J., Minneman K.P. (2005). Recent progress in alpha1-adrenergic receptor research. Acta Pharmacol. Sin..

[47] Kaykı-Mutlu G., Papazisi O., Palmen M., Danser A.H.J., Michel M.C., Arioglu-Inan E. (2020). Cardiac and Vascular α1-Adrenoceptors in Congestive Heart Failure: A Systematic Review. Cells.

[48] Zalewska E., Kmieć P., Sworczak K. (2022). Role of Catestatin in the Cardiovascular System and Metabolic Disorders. Front. Cardiovasc. Med..

[49] Chruscinski A., Brede M.E., Meinel L., Lohse M.J., Kobilka B.K., Hein L. (2001). Differential distribution of beta-adrenergic receptor subtypes in blood vessels of knockout mice lacking beta(1)- or beta(2)-adrenergic receptors. Mol. Pharmacol..

[50] Berlan M., Galitzky J., Bousquet-Melou A., Lafontan M., Montastruc J.L. (1994). Beta-3 adrenoceptor-mediated increase in cutaneous blood flow in the dog. J. Pharmacol. Exp. Ther..

[51] Taylor B.N., Cassagno L.M. (2023). Alpha-Adrenergic Receptors. StatPearls [Internet].

[52] Alhayek S.P.C. (2022). Beta 1 Receptors. StatPearls [Internet].

[53] Vrablik M., Corsini A., Tůmová E. (2022). Beta-blockers for Atherosclerosis Prevention: A Missed Opportunity?. Curr. Atheroscler. Rep..

[54] Baskin A.S., Linderman J.D., Brychta R.J., McGehee S., Anflick-Chames E., Cero C., Johnson J.W., O’Mara A.E., Fletcher L.A., Leitner B.P. (2018). Regulation of Human Adipose Tissue Activation, Gallbladder Size, and Bile Acid Metabolism by a β3-Adrenergic Receptor Agonist. Diabetes.

[55] Larson C.J. (2019). Translational pharmacology and physiology of brown adipose tissue in human disease and treatment. Handb. Exp. Pharmacol..

[56] King A.J., Osborn J.W., Fink G.D. (2007). Splanchnic circulation is a critical neural target in angiotensin II salt hypertension in rats. Hypertension.

[57] Navar L.G. (2014). Physiology: Hemodynamics, endothelial function, renin-angiotensin-aldosterone system, sympathetic nervous system. J. Am. Soc. Hypertens..

[58] Hafen B.B., Shook M., Burns B. (2022). Anatomy, Smooth Muscle. StatPearls [Internet].

[59] Kauffenstein G., Laher I., Matrougui K., Guérineau N.C., Henrion D. (2012). Emerging role of G protein-coupled receptors in microvascular myogenic tone. Cardiovasc. Res..

[60] Somlyo A.P., Somlyo A.V. (2003). Ca2+ sensitivity of smooth muscle and nonmuscle myosin II: Modulated by G proteins, kinases, and myosin phosphatase. Physiol. Rev..

[61] Zicha J., Behuliak M., Vavřínová A., Dobešová Z., Kuneš J., Rauchová H., Vaněčková I. (2021). Cooperation of augmented calcium sensitization and increased calcium entry contributes to high blood pressure in salt-sensitive Dahl rats. Hypertens. Res..

[62] Mueed I., Bains P., Zhang L., Macleod K.M. (2004). Differential participation of protein kinase C and Rho kinase in alpha 1-adrenoceptor mediated contraction in rat arteries. Can. J. Physiol. Pharmacol..

[63] Takashima S.-I., Sugimoto N., Takuwa N., Okamoto Y., Yoshioka K., Takamura M., Takata S., Kaneko S., Takuwa Y. (2008). G12/13 and Gq mediate S1P2-induced inhibition of Rac and migration in vascular smooth muscle in a manner dependent on Rho but not Rho kinase. Cardiovasc. Res..

[64] Espinoza-Derout J., Shao X.M., Lao C.J., Hasan K.M., Rivera J.C., Jordan M.C., Echeverria V., Roos K.P., Sinha-Hikim A.P., Friedman T.C. (2022). Electronic Cigarette Use and the Risk of Cardiovascular Diseases. Front. Cardiovasc. Med..

[65] Shiina S., Kanemura A., Suzuki C., Yamaki F., Obara K., Chino D., Tanaka Y. (2018). β-Adrenoceptor subtypes and cAMP role in adrenaline- and noradrenaline-induced relaxation in the rat thoracic aorta. J. Smooth Muscle Res..

[66] White R.E., Kryman J.P., El-Mowafy A.M., Han G., Carrier G.O. (2000). cAMP-Dependent Vasodilators Cross-Activate the cGMP-Dependent Protein Kinase to Stimulate BKCa Channel Activity in Coronary Artery Smooth Muscle Cells. Circ. Res..

[67] Priest R.M., Hucks D., Ward J.P. (1997). Noradrenaline, beta-adrenoceptor mediated vasorelaxation and nitric oxide in large and small pulmonary arteries of the rat. Br. J. Pharmacol..

[68] Kowala M.C., Nunnari J.J., Durham S.K., Nicolosi R.J. (1991). Doxazosin and cholestyramine similarly decrease fatty streak formation in the aortic arch of hyperlipidemic hamsters. Atherosclerosis.

[69] Swindell A.C., Krupp M.N., Twomey T.M., Reynolds J.A., Chichester C.O. (1993). Effects of doxazosin on atherosclerosis in cholesterol-fed rabbits. Atherosclerosis.

[70] Vashisht R., Sian M., Franks P.J., O’Malley M.K. (1992). Long-term reduction of intimal hyperplasia by the selective alpha-1 adrenergic antagonist doxazosin. Br. J. Surg..

[71] Hoogerbrugge N., de Groot E., de Heide L.H., de Ridder M.A., Birkenhägeri J.C., Stijnen T., Jansen H. (2002). Doxazosin and hydrochlorothiazide equally affect arterial wall thickness in hypertensive males with hypercholesterolaemia (the DAPHNE study). Doxazosin Atherosclerosis Progression Study in Hypertensives in the Netherlands. Neth. J. Med..

[72] Nafstad I., Tollersrud S., Eriksen K., Helgeland A., Solberg L.A., Bredesen J., Dale O. (1988). The influence of atenolol and prazosin on serum lipids and atherosclerosis in minipigs fed a hyperlipidemic diet. Gen. Pharmacol..

[73] Kinoshita M., Shimazu N., Fujita M., Fujimaki Y., Kojima K., Mikuni Y., Horie E., Teramoto T. (2001). Doxazosin, an alpha1-adrenergic antihypertensive agent, decreases serum oxidized LDL. Am. J. Hypertens..

[74] Piller L.B., Davis B.R., Cutler J.A., Cushman W.C., Wright J.T., Williamson J.D., Leenen F.H.H., Einhorn P.T., Randall O.S., Golden J.S. (2002). Validation of Heart Failure Events in the Antihypertensive and Lipid Lowering Treatment to Prevent Heart Attack Trial (ALLHAT) Participants Assigned to Doxazosin and Chlorthalidone. Curr. Control. Trials Cardiovasc. Med..

[75] Durkee C.A., Covelo A., Lines J., Kofuji P., Aguilar J., Araque A. (2019). G(i/o) protein-coupled receptors inhibit neurons but activate astrocytes and stimulate gliotransmission. Glia.

[76] Wang Y., Nguyen D.T., Anesi J., Alramahi A., Witting P.K., Chai Z., Khan A.W., Kelly J., Denton K.M., Golledge J. (2023). Moxonidine Increases Uptake of Oxidised Low-Density Lipoprotein in Cultured Vascular Smooth Muscle Cells and Inhibits Atherosclerosis in Apolipoprotein E-Deficient Mice. Int. J. Mol. Sci..

[77] Al-Sharea A., Lee M.K.S., Whillas A., Michell D.L., Shihata W.A., Nicholls A.J., Cooney O.D., Kraakman M.J., Veiga C.B., Jefferis A.M. (2019). Chronic sympathetic driven hypertension promotes atherosclerosis by enhancing hematopoiesis. Haematologica.

[78] Wang J., Venugopal J., Silaghi P., Su E.J., Guo C., Lawrence D.A., Eitzman D.T. (2023). Beta1-receptor blockade attenuates atherosclerosis progression following traumatic brain injury in apolipoprotein E deficient mice. PLoS ONE.

[79] Ulleryd M.A., Bernberg E., Yang L.J., Bergström G.M., Johansson M.E. (2014). Metoprolol reduces proinflammatory cytokines and atherosclerosis in ApoE^−/−^mice. Biomed. Res. Int..

[80] Chen S.J., Tsui P.F., Chuang Y.P., Chiang D.M., Chen L.W., Liu S.T., Lin F.Y., Huang S.M., Lin S.H., Wu W.L. (2019). Carvedilol Ameliorates Experimental Atherosclerosis by Regulating Cholesterol Efflux and Exosome Functions. Int. J. Mol. Sci..

[81] Shimada K., Hirano E., Kimura T., Fujita M., Kishimoto C. (2012). Carvedilol reduces the severity of atherosclerosis in apolipoprotein E-deficient mice via reducing superoxide production. Exp. Biol. Med..

[82] de Nigris F., Mancini F.P., Balestrieri M.L., Byrns R., Fiorito C., Williams-Ignarro S., Palagiano A., Crimi E., Ignarro L.J., Napoli C. (2008). Therapeutic dose of nebivolol, a nitric oxide-releasing beta-blocker, reduces atherosclerosis in cholesterol-fed rabbits. Nitric Oxide.

[83] Thakur N.K., Hayashi T., Sumi D., Kano H., Matsui-Hirai H., Tsunekawa T., Iguchi A. (2002). Anti-atherosclerotic effect of beta-blocker with nitric oxide-releasing action on the severe atherosclerosis. J. Cardiovasc. Pharmacol..

[84] Hedblad B., Wikstrand J., Janzon L., Wedel H., Berglund G. (2001). Low-dose metoprolol CR/XL and fluvastatin slow progression of carotid intima-media thickness: Main results from the β-Blocker Cholesterol-Lowering Asymptomatic Plaque Study (BCAPS). Circulation.

[85] Wiklund O., Hulthe J., Wikstrand J., Schmidt C., Olofsson S.O., Bondjers G. (2002). Effect of controlled release/extended release metoprolol on carotid intima-media thickness in patients with hypercholesterolemia: A 3-year randomized study. Stroke.

[86] Helgeland A., Eriksen K., Foss P.O., Nafstad I., Solberg L.A., Tollersrud S. (1984). The influence of prazosin and propranolol on serum lipids and atherosclerosis in standard fed pigs. Acta Pharmacol. Toxicol..

[87] van Gestel Y.R., Hoeks S.E., Sin D.D., Welten G.M., Schouten O., Witteveen H.J., Simsek C., Stam H., Mertens F.W., Bax J.J. (2008). Impact of cardioselective beta-blockers on mortality in patients with chronic obstructive pulmonary disease and atherosclerosis. Am. J. Respir. Crit. Care Med..

[88] Shannon A.H., Mehaffey J.H., Cullen J.M., Hawkins R.B., Roy R., Upchurch G.R., Robinson W.P. (2019). Preoperative beta blockade is associated with increased rates of 30-day major adverse cardiac events in critical limb ischemia patients undergoing infrainguinal revascularization. J. Vasc. Surg..

[89] Shavadia J.S., Zheng Y., Green J.B., Armstrong P.W., Westerhout C.M., McGuire D.K., Cornel J.H., Holman R.R., Peterson E.D. (2019). Associations between β-blocker therapy and cardiovascular outcomes in patients with diabetes and established cardiovascular disease. Am. Heart J..

[90] Cimaglia P., Bernucci D., Cardelli L.S., Carone A., Scavone G., Manfrini M., Censi S., Calvi S., Ferrari R., Campo G. (2022). Renin-Angiotensin-Aldosterone System Inhibitors, Statins, and Beta-Blockers in Diabetic Patients With Critical Limb Ischemia and Foot Lesions. J. Cardiovasc. Pharmacol. Ther..

[91] Albiñana V., Recio-Poveda L., González-Peramato P., Martinez-Piñeiro L., Botella L.M., Cuesta A.M. (2022). Blockade of β2-Adrenergic Receptor Reduces Inflammation and Oxidative Stress in Clear Cell Renal Cell Carcinoma. Int. J. Mol. Sci..

[92] Hoeke G., Wang Y., van Dam A.D., Mol I.M., Gart E., Klop H.G., van den Berg S.M., Pieterman E.H., Princen H.M.G., Groen A.K. (2017). Atorvastatin accelerates clearance of lipoprotein remnants generated by activated brown fat to further reduce hypercholesterolemia and atherosclerosis. Atherosclerosis.

[93] Zhou E., Li Z., Nakashima H., Liu C., Ying Z., Foks A.C., Berbée J.F.P., van Dijk K.W., Rensen P.C.N., Wang Y. (2021). Hepatic Scavenger Receptor Class B Type 1 Knockdown Reduces Atherosclerosis and Enhances the Antiatherosclerotic Effect of Brown Fat Activation in APOE*3-Leiden.CETP Mice. Arterioscler. Thromb. Vasc. Biol..

[94] Zhou E., Hoeke G., Li Z., Eibergen A.C., Schonk A.W., Koehorst M., Boverhof R., Havinga R., Kuipers F., Coskun T. (2020). Colesevelam enhances the beneficial effects of brown fat activation on hyperlipidaemia and atherosclerosis development. Cardiovasc. Res..

[95] Zhou E., Li Z., Nakashima H., Choukoud A., Kooijman S., Berbée J.F.P., Rensen P.C.N., Wang Y. (2021). Beneficial effects of brown fat activation on top of PCSK9 inhibition with alirocumab on dyslipidemia and atherosclerosis development in APOE*3-Leiden.CETP mice. Pharmacol. Res..

[96] O’Mara A.E., Johnson J.W., Linderman J.D., Brychta R.J., McGehee S., Fletcher L.A., Fink Y.A., Kapuria D., Cassimatis T.M., Kelsey N. (2020). Chronic mirabegron treatment increases human brown fat, HDL cholesterol, and insulin sensitivity. J. Clin. Investig..

[97] Cypess A.M., Weiner L.S., Roberts-Toler C., Franquet Elía E., Kessler S.H., Kahn P.A., English J., Chatman K., Trauger S.A., Doria A. (2015). Activation of human brown adipose tissue by a β3-adrenergic receptor agonist. Cell Metab..

[98] Malik M., van Gelderen E.M., Lee J.H., Kowalski D.L., Yen M., Goldwater R., Mujais S.K., Schaddelee M.P., de Koning P., Kaibara A. (2012). Proarrhythmic safety of repeat doses of mirabegron in healthy subjects: A randomized, double-blind, placebo-, and active-controlled thorough QT study. Clin. Pharmacol. Ther..

[99] Li L., Xiong Y.L., Tu B., Liu S.Y., Zhang Z.H., Hu Z., Yao Y. (2023). Effect of renal denervation for patients with isolated systolic hypertension: A systematic review and meta-analysis. J. Geriatr. Cardiol..

[100] Huang H.C., Cheng H.M., Chia Y.C., Li Y., Van Minh H., Siddique S., Sukonthasarn A., Tay J.C., Turana Y., Verma N. (2022). The role of renal nerve stimulation in percutaneous renal denervation for hypertension: A mini-review. J. Clin. Hypertens..

[101] Rey-García J., Townsend R.R. (2022). Renal Denervation: A Review. Am. J. Kidney Dis..

[102] Wang Y., Seto S.W., Golledge J. (2013). Therapeutic effects of renal denervation on renal failure. Curr. Neurovasc. Res..

[103] Schmieder R.E. (2023). Renal denervation in patients with chronic kidney disease: Current evidence and future perspectives. Nephrol. Dial. Transplant..

[104] Nawar K., Mohammad A., Johns E.J., Abdulla M.H. (2023). Renal denervation for atrial fibrillation: A comprehensive updated systematic review and meta-analysis. J. Hum. Hypertens..

[105] Li Z., Li Q., Wang L., Li C., Xu M., Duan Y., Ma L., Li T., Chen Q., Wang Y. (2021). Targeting mitochondria-inflammation circle by renal denervation reduces atheroprone endothelial phenotypes and atherosclerosis. Redox Biol..

[106] Chen H., Wang R., Xu F., Zang T., Ji M., Yin J., Chen J., Shen L., Ge J. (2020). Renal denervation mitigates atherosclerosis in ApoE^−/−^mice via the suppression of inflammation. Am. J. Transl. Res..

[107] Wang H., Wang J., Guo C., Luo W., Kleiman K., Eitzman D.T. (2015). Renal denervation attenuates progression of atherosclerosis in apolipoprotein E-deficient mice independent of blood pressure lowering. Hypertension.

[108] Visentin V., Boucher J., Bour S., Prévot D., Castan I., Carpéné C., Valet P. (2005). Influence of high-fat diet on amine oxidase activity in white adipose tissue of mice prone or resistant to diet-induced obesity. J. Physiol. Biochem..

[109] Wang Y., Dinh T.N., Nield A., Krishna S.M., Denton K., Golledge J. (2017). Renal Denervation Promotes Atherosclerosis in Hypertensive Apolipoprotein E-Deficient Mice Infused with Angiotensin II. Front. Physiol..

[110] Su E., Zhao L., Yang X., Zhu B., Liu Y., Zhao W., Wang X., Qi D., Zhu L., Gao C. (2020). Aggravated endothelial endocrine dysfunction and intimal thickening of renal artery in high-fat diet-induced obese pigs following renal denervation. BMC Cardiovasc. Disord..

[111] Kuzuya M., Nakamura K., Sasaki T., Cheng X.W., Itohara S., Iguchi A. (2006). Effect of MMP-2 deficiency on atherosclerotic lesion formation in apoE-deficient mice. Arterioscler. Thromb. Vasc. Biol..

[112] Kosowski M., Basiak M., Hachuła M., Okopień B. (2022). Plasma Concentrations of New Biochemical Markers of Atherosclerosis in Patients with Dyslipidemia-A Pilot Study. Medicina.

[113] Tu Y., Ma X., Chen H., Fan Y., Jiang L., Zhang R., Cheng Z. (2022). Molecular Imaging of Matrix Metalloproteinase-2 in Atherosclerosis Using a Smart Multifunctional PET/MRI Nanoparticle. Int. J. Nanomed..

[114] D’Orléans-Juste P., Plante M., Honoré J.C., Carrier E., Labonté J. (2003). Synthesis and degradation of endothelin-1. Can. J. Physiol. Pharmacol..

[115] Abdalvand A., Morton J.S., Bourque S.L., Quon A.L., Davidge S.T. (2013). Matrix metalloproteinase enhances big-endothelin-1 constriction in mesenteric vessels of pregnant rats with reduced uterine blood flow. Hypertension.

[116] Chen S., Mukherjee S., Chakraborty C., Chakrabarti S. (2003). High glucose-induced, endothelin-dependent fibronectin synthesis is mediated via NF-kappa B and AP-1. Am. J. Physiol. Cell Physiol..

[117] Yu H., Alruwaili N., Kelly M.R., Zhang B., Liu A., Wang Y., Sun D., Wolin M.S. (2022). Endothelin-1 depletion of cartilage oligomeric matrix protein modulates pulmonary artery superoxide and iron metabolism-associated mitochondrial heme biosynthesis. Am. J. Physiol. Lung Cell Mol. Physiol..

[118] Shoeb M., Ansari N.H., Srivastava S.K., Ramana K.V. (2014). 4-Hydroxynonenal in the pathogenesis and progression of human diseases. Curr. Med. Chem..

[119] Mahfoud F., Mancia G., Schmieder R.E., Ruilope L., Narkiewicz K., Schlaich M., Williams B., Ribichini F., Weil J., Almerri K. (2023). Outcomes Following Radiofrequency Renal Denervation According to Antihypertensive Medications: Subgroup Analysis of the Global SYMPLICITY Registry DEFINE. Hypertension.

[120] Barbato E., Azizi M., Schmieder R.E., Lauder L., Böhm M., Brouwers S., Bruno R.M., Dudek D., Kahan T., Kandzari D.E. (2023). Renal denervation in the management of hypertension in adults. A clinical consensus statement of the ESC Council on Hypertension and the European Association of Percutaneous Cardiovascular Interventions (EAPCI). Eur. Heart J..

[121] Wang Y. (2014). Renal denervation for resistant hypertension—The Symplicity HTN-1 study. Lancet.

[122] Wang Y., Lim K., Denton K.M. (2017). Editorial: Function of Renal Sympathetic Nerves. Front. Physiol..

[123] Wang Y. (2014). What is the true incidence of renal artery stenosis after sympathetic denervation?. Front. Physiol..

[124] Wang Y. (2014). Single-sided renal denervation may be not suitable for patients with significant renal artery stenosis. Clin. Res. Cardiol..

[125] Wang Y. (2014). It may be not suitable to perform renal denervation in renal arteries with significant stenosis. Int. J. Cardiol..

[126] Wang Y. (2015). Does Renal Denervation Inhibit Atherosclerosis in Humans?. Austin J. Cardiovasc. Dis. Atheroscler..

[127] Wang Y. (2014). Patients with renal artery stenosis may not be suitable for renal denervation. Clin. Res. Cardiol..

[128] Wang Y. (2014). Renal artery stenosis may be responsible for the gradual return of high blood pressure after renal denervation. J. Clin. Hypertens.

[129] Dorr O., Liebetrau C., Mollmann H., Mahfoud F., Ewen S., Gaede L., Troidl C., Hoffmann J., Busch N., Laux G. (2015). Beneficial effects of renal sympathetic denervation on cardiovascular inflammation and remodeling in essential hypertension. Clin. Res. Cardiol..

[130] Dhillon O.S., Khan S.Q., Narayan H.K., Ng K.H., Mohammed N., Quinn P.A., Squire I.B., Davies J.E., Ng L.L. (2009). Matrix metalloproteinase-2 predicts mortality in patients with acute coronary syndrome. Clin. Sci..

[131] Zhang Z.H., Yang K., Jiang F.L., Zeng L.X., Jiang W.H., Wang X.Y. (2014). The effects of catheter-based radiofrequency renal denervation on renal function and renal artery structure in patients with resistant hypertension. J. Clin. Hypertens..

[132] Murphy T.O., Haglin J.J., Felder D.A. (1957). The progression of experimental atherosclerosis after lumbar sympathectomy. Surg. Forum.

[133] Criqui M.H., Aboyans V. (2015). Epidemiology of Peripheral Artery Disease. Circ. Res..

[134] Canani L.H., Copstein E., Pecis M., Friedman R., Leitão C.B., Azevedo M.J., Triches C., Rados D.R., Moreas R.S., Gross J.L. (2013). Cardiovascular autonomic neuropathy in type 2 diabetes mellitus patients with peripheral artery disease. Diabetol. Metab. Syndr..

[135] Qin L., Cui J., Li J. (2022). Sympathetic Nerve Activity and Blood Pressure Response to Exercise in Peripheral Artery Disease: From Molecular Mechanisms, Human Studies, to Intervention Strategy Development. Int. J. Mol. Sci..

[136] Li J., Xing J. (2012). Muscle afferent receptors engaged in augmented sympathetic responsiveness in peripheral artery disease. Front. Physiol..

[137] Qin L., Li J. (2021). Nerve growth factor in muscle afferent neurons of peripheral artery disease and autonomic function. Neural Regen. Res..

[138] Chowdhury M., Nevitt S., Eleftheriadou A., Kanagala P., Esa H., Cuthbertson D.J., Tahrani A., Alam U. (2021). Cardiac autonomic neuropathy and risk of cardiovascular disease and mortality in type 1 and type 2 diabetes: A meta-analysis. BMJ Open Diabetes Res. Care.

[139] Astrup A.S., Tarnow L., Rossing P., Hansen B.V., Hilsted J., Parving H.H. (2006). Cardiac autonomic neuropathy predicts cardiovascular morbidity and mortality in type 1 diabetic patients with diabetic nephropathy. Diabetes Care.

